# Phenotypic and Molecular Characterization of *Naganishia uzbekistanensis*: Diagnostic Challenges and Antifungal Resistance Profile

**DOI:** 10.1002/jcla.70251

**Published:** 2026-05-02

**Authors:** Xin Fan, Xinfei Chen, Jin Li, Rongchen Dai, Pengjie Hu, Li Gu, Meng Xiao, Xiaomin Yu

**Affiliations:** ^1^ Department of Infectious Diseases and Clinical Microbiology Beijing Institute of Respiratory Medicine and Beijing Chao‐Yang Hospital, Capital Medical University Beijing China; ^2^ Department of Laboratory Medicine Peking Union Medical College Hospital, Chinese Academy of Medical Sciences and Peking Union Medical College Beijing China; ^3^ State Key Laboratory of Mycology Institute of Microbiology, Chinese Academy of Sciences Beijing China

**Keywords:** antifungal resistance, clinical mycology, laboratory identification, *Naganishia uzbekistanensis*, scanning electron microscopy

## Abstract

**Background:**

*Naganishia (N.) uzbekistanensis* is a rare basidiomycetous yeast within the family *Filobasidiaceae*, widely distributed in environmental niches, with very limited clinical data available. Its clinical and microbiological features remain poorly understood, posing diagnostic challenges in clinical microbiology laboratories.

**Methods:**

We describe a pulmonary infection caused by *N. uzbekistanensis* strain CY11558 and present an expanded laboratory investigation of this previously reported clinical strain. The strain was analyzed using a comprehensive laboratory workflow that included conventional culture, light microscopy, scanning electron microscopy (SEM), and molecular identification based on rDNA sequencing. Phylogenetic analysis was conducted to assess the genetic relationship with reference strains. Antifungal susceptibility testing and in vitro Titan cell induction assays were also performed.

**Results:**

Conventional MALDI‐TOF MS failed to identify the strain, while rDNA sequencing confirmed *N. uzbekistanensis*. Cryptococcal antigen testing yielded a weakly positive result with the pure culture supernatant, while the patient's serum remained negative. The strain showed markedly elevated MICs for echinocandins, flucytosine, and fluconazole, whereas MICs for voriconazole, itraconazole, and amphotericin B were comparatively low. Microscopic examination revealed round yeast cells without pseudohyphae. SEM showed globular cells enveloped by a fibrillar network. Titan cell formation was not observed. Phylogenetic analysis demonstrated intraspecies variability without geographic association.

**Conclusions:**

This study provides comprehensive phenotypic, including antifungal susceptibility, and molecular characterization of *N. uzbekistanensis*, emphasizing the need for molecular tools in identifying uncommon yeasts. The findings expand current understanding of its morphological and resistance traits and highlight potential diagnostic challenges in clinical laboratories.

## Graphical abstract

The graphical abstract summarizes the laboratory characterization of *Naganishia uzbekistanensis* strain CY11558. The isolate shows distinct colony morphology, globular yeast cells with a fibrillar surface network under scanning electron microscopy, and a weakly positive cryptococcal antigen reaction. No Titan cell formation was observed under in vitro induction conditions.

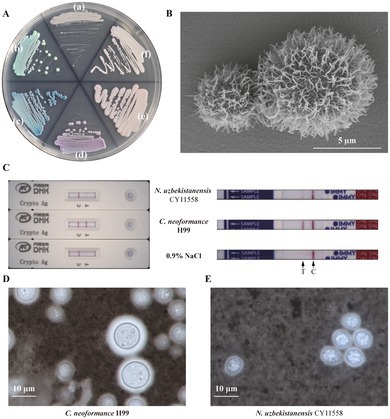

## 1 Introduction

Opportunistic fungal pathogens have become a growing concern nowadays, particularly among immunocompromised hosts [1, 2]. While *Cryptococcus* and *Candida* species remain the most prevalent fungal pathogens, rare and emerging yeasts are increasingly recognized as causes of life‐threatening infections and pose significant diagnostic and therapeutic challenges [1].

The genus *Naganishia* belongs to the family Filobasidiaceae. It was formerly included within the polyphyletic genus *Cryptococcus* and currently comprises over 20 species [3]. To date, the majority of *Naganishia* species have been recovered from environmental sources such as soil and water [3]. Notably, some *Naganishia* species survive in extreme environments, including high‐altitude regions and even from the space station [3, 4], which has drawn ongoing scientific attention to this genus. However, with the exception of *Naganishia albida*, other *Naganishia* species have rarely been reported as causative agents of human infections.

*Naganishia* (*N.) uzbekistanensis* was first identified from a soil sample collected in Uzbekistan in 1999, and has generally been regarded as an environmental saprophyte [5, 6]. To date, very few clinical infections caused by *N. uzbekistanensis* have been described in the literature. Powel et al. [6] reported a severe bone marrow infection in 2012. Despite receiving high‐dose fluconazole therapy for the bone marrow infection, the patient's condition continued to deteriorate, ultimately resulting in death. In addition, genomic data derived from the same clinical strain analyzed in the present study have been previously published by our group [7]. Given its relative rarity and the limited available data, further exploration is warranted. In the present study, we provide an expanded clinical, microbiological, and laboratory characterization of the same clinical strain through a follow‐up and in‐depth investigation.

## 2 Case Description

A 58‐year‐old female was admitted to the thoracic surgery ward on May 26th, 2022, with the complaint of right lower lung ground‐glass opacity found on physical examination for one and a half years. On May 30th (Day 0), wedge resection of the lower lobe of the right lung via video‐assisted thoracoscopic surgery (VATS) was performed, revealing minimally invasive adenocarcinoma on postoperative pathology.

On June 1st (postoperative Day 2), the patient developed fever (maximum temperature 38.0°C), accompanied by cough and hemoptysis. A repeat chest CT demonstrated patchy exudative opacities localized to the residual right lower lobe. Routine blood tests revealed elevated white blood cell (WBC) count of 10.72 × 10^9^/L with 84.0% neutrophils, and C‐reactive protein (CRP) was elevated to 65.6 mg/L. Blood culture, pleural fluid culture, and serum cryptococcal capsular antigen test results were negative. Empiric antibacterial therapy (moxifloxacin 0.4 g IV qd) was initiated, yet the patient's fever persisted and hemoptysis worsened without clinical improvement.

The patient underwent resection of the residual portion of the right lower lobe on June 7th (Day 8). Intraoperatively, a localized hematoma was observed at the incisional margin of the right lower lobe. Histopathological examination of the resected specimen revealed localized necrosis and hemorrhage of lung tissue, increased neutrophilic exudation, and formation of small abscesses. After the operation, moxifloxacin therapy was continued but the patient's fever persisted.

The patient's excised lung tissue underwent routine microbiological examination. No bacterial pathogen was isolated on Columbia blood, Chocolate, or China blue lactose agar (Thermo Fisher Scientific, USA), while positive yeast growth was observed on both Sabouraud dextrose (Oxoid, UK) and CHROMagar *Candida* (CHROMagar, France) agars. The MALDI‐TOF MS system (VITEK MS, bioMérieux, France) failed to identify the strain. Later, the strain was confirmed as *N. uzbekistanensis* through rDNA Sanger sequencing (see Methods). Pending accurate species identification, empirical antifungal therapy with intravenous caspofungin was initiated on Day 12, under the presumption of *Candida* infection. After 7 days, the regimen was transitioned on June 18 (Day 19) to oral voriconazole (200 mg bid), which was continued thereafter. The patient's fever gradually resolved over the subsequent days.

**FIGURE 1:**
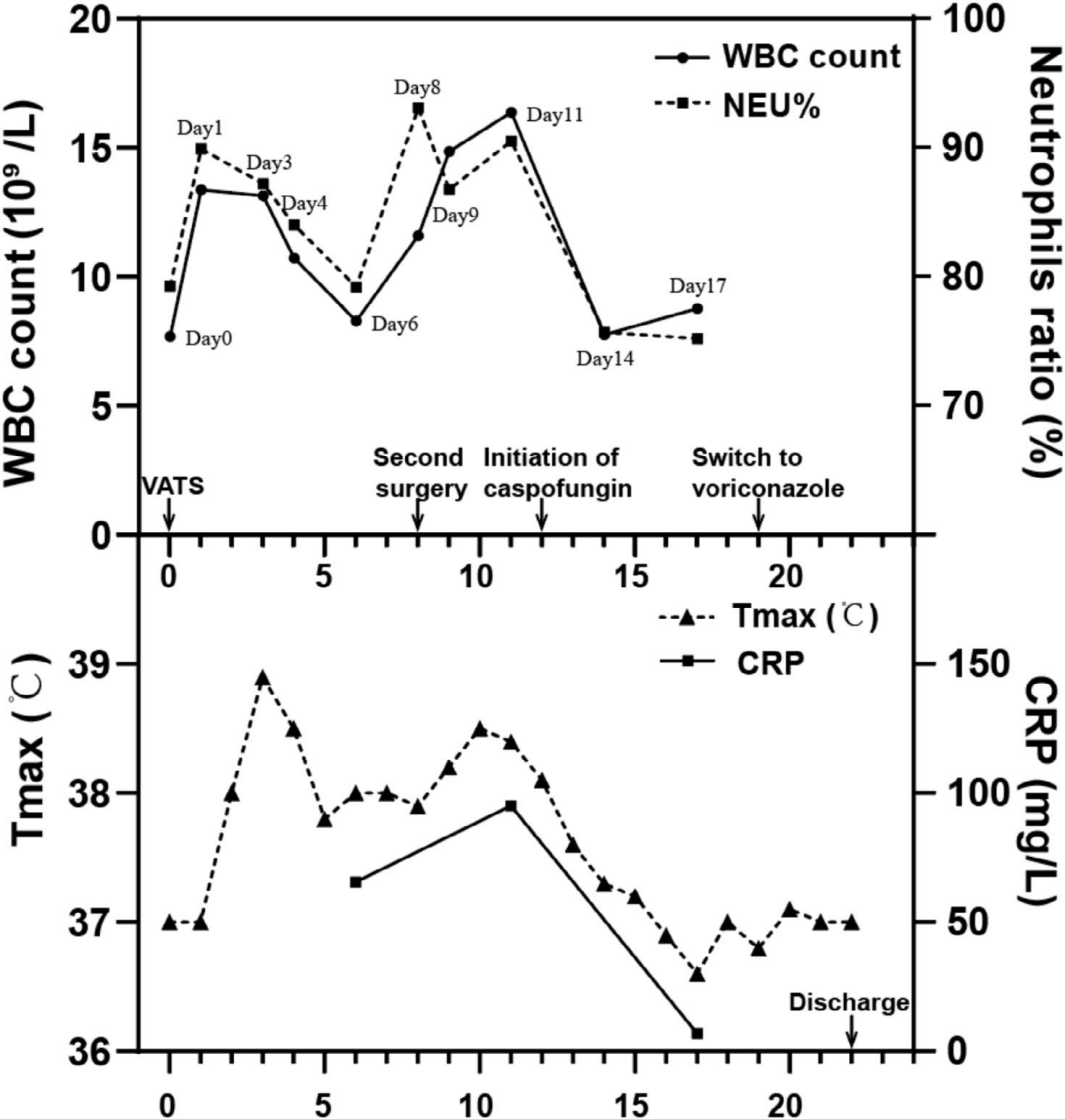
Patient's clinical course and key interventions. CRP, C‐reactive protein; NEU, neutrophil; *T*_max_, maximum body temperature; VATS, video‐assisted thoracoscopic surgery; WBC, white blood cell.

Given the phylogenetic relatedness of *N. uzbekistanensis* to the *Cryptococcus* genus, serum cryptococcal capsular antigen testing was repeated on June 16 (Day 17), but the results were negative. A follow‐up lung CT on June 20 (Day 21) indicated a marked reduction in inflammatory consolidation of the right lower lobe compared to prior imaging. Concurrently, the patient's blood leukocyte counts and CRP levels normalized, with a WBC count of 8.77 × 10^9^/L and 75.2% neutrophils, and CRP at 6.93 mg/L. She was discharged from the hospital in stable condition on June 22. Patient's clinical course and key interventions were summarized in Figure 1.

Following discharge, the patient continued oral voriconazole at 200 mg twice daily for 7 days, and was then changed to itraconazole at 200 mg twice daily during outpatient follow‐up at another local outpatient clinic, completing a total of 3 months of therapy. A subsequent lung CT scan in March 2023 revealed complete resolution of the pulmonary lesion.

## 3 Methods

### 3.1 Strain Description

The *N. uzbekistanensis* strain analyzed in the present study was the same clinical strain previously reported by our group, for which a draft genome sequence was described [7]. In the current study, this strain was further analyzed for antifungal susceptibility, morphological characteristics, and laboratory diagnostic features.

### 3.2 Phenotypic Examinations

The clinical *N. uzbekistanensis* strain CY11558 obtained from the patient was subjected to further investigations. Morphology of colonies was examined after culturing at 28°C for 48 h on Sabouraud dextrose agar (SDA) and CHROMagar *Candida* chromogenic media. Microscopic examinations were performed after overnight growth of the strain in yeast extract‐peptone‐dextrose (YPD) broth using an optical and a scanning electron microscope (SU8000, Hitachi Ltd., Japan), operated at 3.0 kV as described in a prior report [8].

### 3.3 Cryptococcal Capsular Polysaccharide Antigen Testing

Given the close phylogenetic relationship between the genus *Naganishia* and *Cryptococcus*, the cryptococcal capsular polysaccharide antigen testing was performed for *N. uzbekistanensis* strain CY11558. Fresh yeast cultures were suspended in sterile physiological saline, adjusted to a turbidity of 0.5 McFarland, and used directly for testing with two commercially available cryptococcal antigen (CrAg) lateral flow assay strips (IMMY, USA and Dynamiker, China), following the manufacturers' instructions. Patient serum samples were tested directly without dilution or additional pretreatment. *Cryptococcus (C.) neoformans* H99 was included as the positive control, while physiological saline served as the negative control.

### 3.4 MALDI‐TOF MS Identification

Species identification was performed using MALDI‐TOF MS (VITEK MS, bioMérieux, France) with the clinical IVD database (version 3.2.0), following the manufacturer's standard operating procedures. Briefly, fresh yeast cultures were directly smeared to the target plate, overlaid with 0.5 μL of formic acid for on‐plate protein extraction, air‐dried, and subsequently covered with 1 μL of α‐cyano‐4‐hydroxycinnamic acid (CHCA) matrix solution. Spectra were acquired automatically and interpreted using the VITEK MS system software, which applies a color coded scoring system to indicate spectrum quality and identification reliability. Only the manufacturer‐provided clinical IVD database was used for analysis, without querying Research Use Only (RUO) or any in‐house spectral libraries.

### 3.5 Molecular Identification and rDNA‐Based Phylogenetic Analysis

Molecular identification and phylogenetic analysis were carried out as previously described [9, 10, 11]. In general, the rDNA internal transcribed spacer (ITS) region and D1/D2 domain of 28S rDNA were amplified [10] and subjected to Sanger sequencing, which was performed at RuiBiotech (Beijing, China) following standard protocols. The resulting sequences were queried against the NCBI RefSeq database (https://www.ncbi.nlm.nih.gov/refseq/targetedloci). Furthermore, rDNA sequences from type strains of all *Naganishia* species publicly available in GenBank (https://www.ncbi.nlm.nih.gov/genbank) were retrieved. In addition, sequences of all available *N. uzbekistanensis* strains (10 strains in all, up to October 31, 2025), were incorporated into the comparative analysis as well. Phylogenetic analysis was performed using Mega X software (version 10.2) by the neighbor‐joining method with bootstrap value of 1000 [11]. The sequence of *Filobasidium wieringae* type strain CBS 1937 T was used as an outgroup for the phylogenetic analysis of the *Naganishia* genus, while the sequence of *N. albida* CBS 142T served as an outgroup for the phylogenetic analysis of *N. uzbekistanensis* strains from different origins.

### 3.6 Antifungal Susceptibility Testing

Minimum inhibitory concentrations (MICs) of *N. uzbekistanensis* strain CY11558 against nine antifungal agents, including anidulafungin, micafungin, caspofungin, flucytosine, itraconazole, voriconazole, posaconazole, fluconazole, and amphotericin B, were determined using the Sensititre YeastOne YO10 commercial kit (Thermo Scientific, USA) according to the manufacturer's instructions. Results were recorded after 72 h of incubation, and *Candida krusei* ATCC 6258 and *Candida parapsilosis* ATCC 22019 were used as quality controls. Because no clinical breakpoints or epidemiological cutoff values have been established for *N. uzbekistanensis*, these MIC results are presented without categorical interpretation.

### 3.7 Titan Cell Induction

Titan cells were induced using two previously described methods [12, 13], with minor adaptations. Briefly, yeast cells were recovered on SDA (30°C, 2–5 days), and approximately 1 × 10^7^ cells were inoculated into 10 mL YPD and incubated at 30°C, 150 rpm for 22 h. After washing twice with minimal medium (MM), 1 × 10^6^ cells were resuspended in 1 mL MM (pH 5.5) and incubated in 800 rpm 72 h for Titan induction. In parallel, overnight cultures grown in YNB with 2% glucose (30°C, 150 rpm) were transferred into 10% HI‐FCS and incubated at 30°C in 5% CO_2_ for 72 h. Cell size was measured by India ink staining. *C. neoformans* H99 was used as a positive control. Cells with diameter of > 10 μm were considered Titan cells [3, 13].

### 3.8 Review of Published Data

Previous publications on *N. uzbekistanensis* were mined by querying the species name “*N. uzbekistanensis*” or its basionym “*Cryptococcus uzbekistanensis*” in PubMed (https://pubmed.ncbi.nlm.nih.gov). Mycobank and Google Scholar (https://scholar.google.com) results were reviewed manually to exclude irrelevant contents. Public databases including GenBank and Mycobank were used for accessing additional *N. uzbekistanensis* strains and other *Naganishia* species. Information was summarized, including strains' geographic origin and source of isolation and available gene sequences.

## 4 Results

**FIGURE 2:**
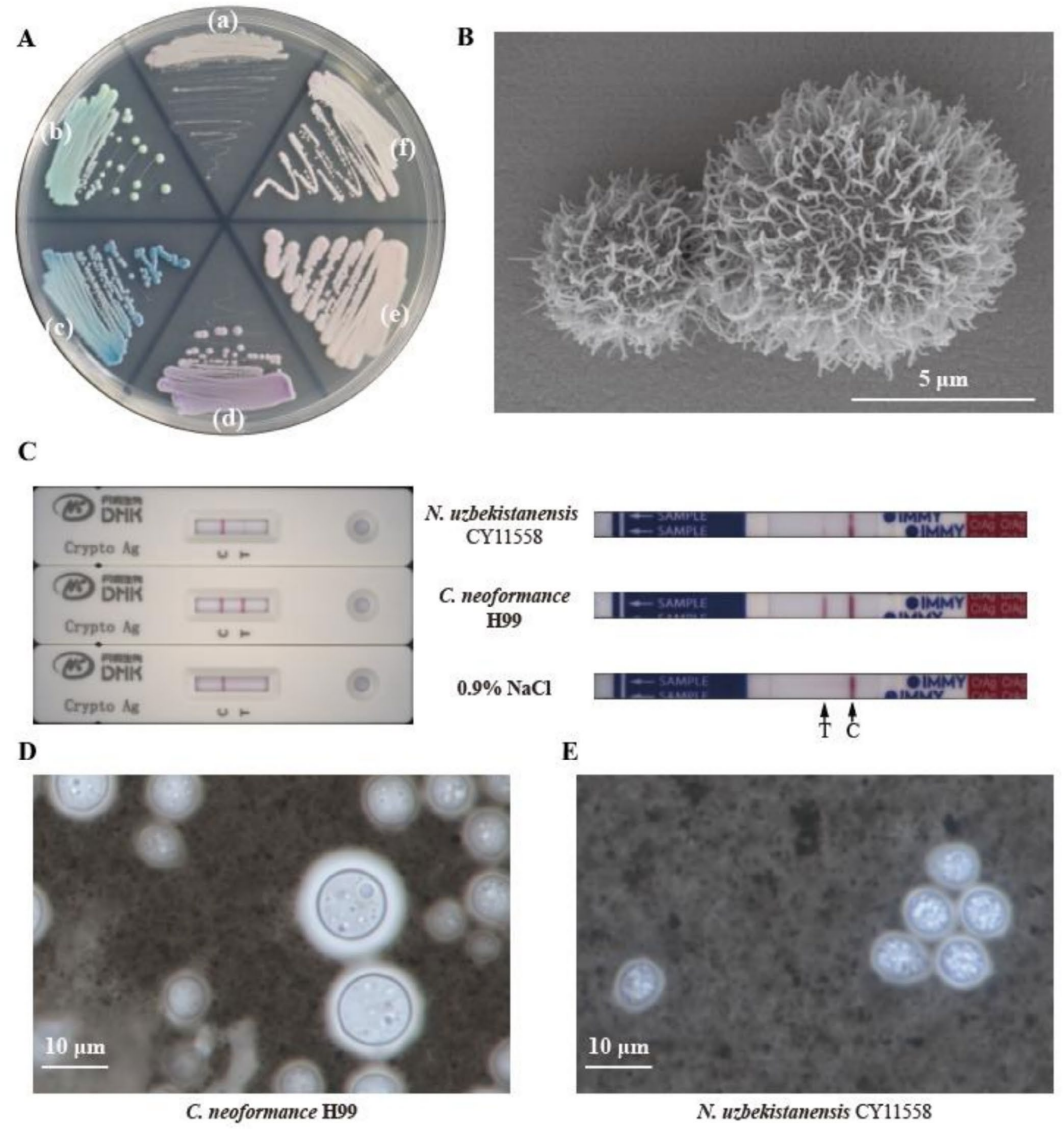
Characteristics of the clinical *N. uzbekistanensis* strain identified in this study. (A) Colony morphology of *N. uzbekistanensis* strain CY11558 compared with those of common *Candida* species on CHROMagar *Candida* after 48 h at 28°C. Strains included were (a) *N. uzbekistanensis* CY11558; (b) *C. albicans* ATCC 90028; (c) *C. tropicalis* ATCC 750; (d) *C. glabrata* clinical strain CY9669; (e) *C. krusei* ATCC 6258; (f) *C. parapsilosis* ATCC 22019. (B) *N. uzbekistanensis* cells of strain CY11558 under scanning electron microscope. (C) The cryptococcal capsular polysaccharide antigen test results for *N. uzbekistanensis* strain CY11558 were weak‐positive. (D, E) In vitro induction of Titan cells. (D) Titan cells (diameter > 10 μm) observed in *C. neoformans* strain H99. (E) No Titan cells were observed in *N. uzbekistanensis* strain CY11558 under the same condition.

After incubating at 28°C for 48 h, the colonies of *N. uzbekistanensis* strain CY11558 on SDA were white, convex, and moist with smooth margins. When grown on CHROMagar *Candida* medium, the *N. uzbekistanensis* strain CY11558 produced small, white colonies without any species‐specific pigmentation (Figure 2A). Under optical microscopy, the yeast cells appeared predominantly spherical, occurring singly or in pairs, with no evidence of pseudohyphal development. Scanning electron microscopy (SEM) revealed globular cells surrounded by a fibrillar network, and the polysaccharide fibrils in some yeasts entwined with adjacent fibrils (Figure 2B). Subsequent species identification using MALDI‐TOF MS was attempted; although spectra acquisition was successful and passed quality control (green), database matching yielded “no identification”, as reference spectra for *N. uzbekistanensis* are not available in the commercial databases.

Two different commercial CrAg lateral flow assay kits were used to detect cryptococcal capsular polysaccharide antigen. The patient's serum sample tested negative with both kits. However, when the yeast suspension from *N. uzbekistanensis* strain CY11558 was directly tested, a weak‐positive result was obtained (Figure 2C).

**FIGURE 3:**
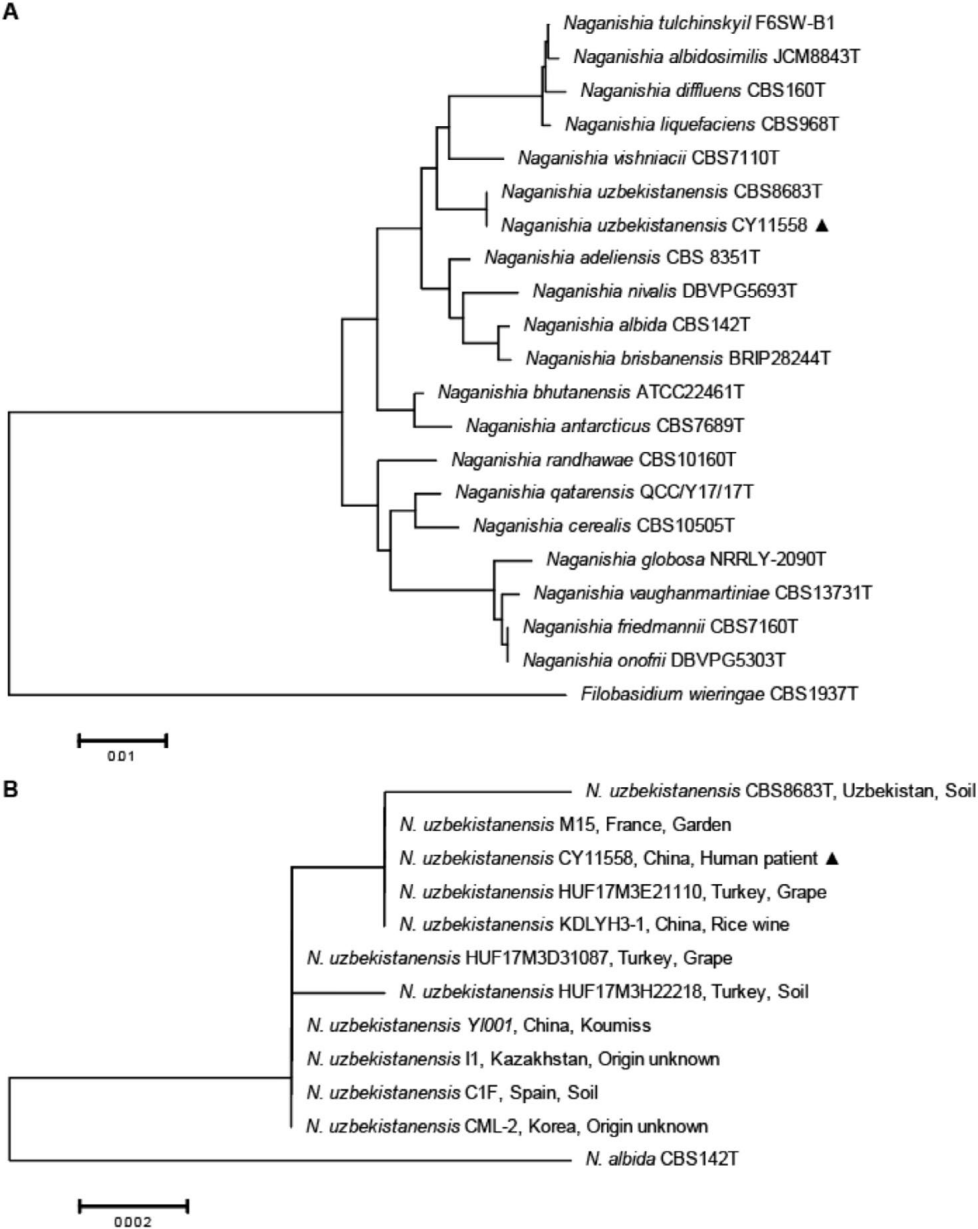
Phylogenetic analysis of *N. uzbekistanensis* clinical strain CY11558 using the neighbor‐joining method. (A) Phylogenetic tree based on concatenated ITS region and D1/D2 domain sequences, constructed with type strains of various *Naganishia* species. (B) Phylogenetic analysis based on ITS region sequences of *N. uzbekistanensis* strains publicly available in the NCBI GenBank database.

The ITS region sequence of the strain (GenBank accession no. PQ187063.1) was 99% (619/621) identical to that of *N. uzbekistanensis* strain CBS8683 (GenBank accession no. NR_073219.1), while the D1/D2 domain of the strain (GenBank accession no. PQ187064.1) showed 100% (628/628) identity with that of strain CBS8683 (GenBank accession no. NG_067254.1) (Figure 3A). Neighbor‐joining phylogenetic analysis revealed intraspecies variations of ITS region sequences within *N. uzbekistanensis*, but the strains' phylogeny was not associated with their geographic origin or source of isolation (Figure 3B).

Antifungal susceptibility testing of *N. uzbekistanensis* strain CY11558 showed markedly elevated MICs for the echinocandins and flucytosine (anidulafungin > 8 μg/mL, micafungin > 8 μg/mL, caspofungin 8 mg/L, and flucytosine > 64 μg/mL). For the remaining antifungal agents, the MIC values were as follows: posaconazole 0.5 μg/mL, voriconazole 0.25 μg/mL, itraconazole 0.25 μg/mL, fluconazole 32 μg/mL, and amphotericin B 1 μg/mL.

A previous study reported that one *Naganishia* species, *N. tulchinskyi*, was capable of forming Titan cells‐an enlarged yeast morphology previously thought to be unique to the *C. neoformans/Cryptococcus gattii* species complex [3]. Based on this observation, we investigated whether titanization could also occur in our *N. uzbekistanensis* strain CY11558. Using two in vitro induction assays previously established [12, 13], we successfully observed Titan cells (diameter > 10 μm) in the positive control strain *C. neoformans* H99 (Figure 2D). In contrast, cells of *N. uzbekistanensis* strain CY11558 ranged in size from 5 to 7 μm, with no Titan cells identified (Figure 2E).

## 5 Discussion

*N. uzbekistanensis* is an exceedingly rare human pathogen, for which clinical experience and microbiological characterization remain extremely limited. Other *Naganishia* species, such as *N. albida* (syn. *Cryptococcus albidus* ), occur more frequently and are known to cause infections (e.g., fungemia, meningitis, and skin lesions) in immunocompromised individuals [14, 15, 16, 17, 18]. The present study provides further clinical and microbiological characterization of this rare organism based on the same previously reported clinical strain, highlighting its potential clinical significance and the diagnostic challenges.

*N. uzbekistanensis* strain CY11558 exhibits slower growth compared with common yeasts, and no hyphae or pseudohyphae were observed, consistent with previous descriptions of this species [5]. SEM revealed globular cells encased in a fibrillar network, resembling the morphology reported in *Cryptococcus* species [19].

Accurate identification of *N. uzbekistanensis* remains challenging. MALDI‐TOF MS did not identify *N. uzbekistanensis* strain CY11558, because reference spectra for this species are not included in current databases. As reported for other uncommon yeasts, DNA sequencing is often required to achieve reliable species‐level identification [1]. Sequencing of the ITS and D1/D2 regions enabled definitive identification in this case, and phylogenetic analysis clearly separated CY11558 from closely related taxa (Figure 3A), underscoring the importance of sequencing‐based diagnostic approaches for rare fungal pathogens [1].

The CrAg test presented another diagnostic challenge. Although serum CrAg tests were negative, the pure culture supernatant yielded weak‐positive results. In infection cases caused by *N. albida* , the most commonly identified human pathogenic species within the genus *Naganishia*, serum CrAg tests are usually negative; however, a positive serum test has also been documented [17]. In this study, CrAg testing showed negative results in patient serum but weak‐positive reactivity in culture suspension. This discrepancy may be due to a lower antigen burden in serum compared with culture suspension, allowing weak cross‐reactivity to be detected only in culture‐based testing. These findings suggest that CrAg assays may exhibit variable reactivity when applied to *Naganishia* species, potentially related to cross‐reactivity. Similar patterns have been reported in other non‐*Cryptococcus* yeasts, such as *Trichosporon* species [20].

Antifungal susceptibility testing of *N. uzbekistanensis* strain CY11558 revealed a pattern consistent with other *Naganishia* species [21]. Elevated MICs were observed against echinocandins and flucytosine, suggesting possible intrinsic resistance to these agents, whereas susceptibility to polyene and most azole antifungals was preserved. Similar to antifungal patterns reported for other *Naganishia* species, the *N. uzbekistanensis* strain exhibited low MICs for itraconazole and voriconazole, whereas its fluconazole MIC was comparatively high [22]. This susceptibility profile is distinct from that of more common yeasts and has direct implications for empirical antifungal therapy.

In the present case, although the patient initially received caspofungin and the strain exhibited markedly elevated MICs to echinocandins, the observed clinical improvement occurred in the context of surgical removal of the infected lesion. Host‐related factors, including immune status, may also have contributed to the clinical course, although their relative impact cannot be determined from this single‐case observation. Following definitive identification, antifungal therapy was transitioned to voriconazole, with continued recovery. Amphotericin B demonstrated low MICs in vitro, indicating that it may serve as an alternative therapeutic option in severe or disseminated infections caused by this species.

Currently, little is known about the virulence factors and ecological adaptations enabling *N. uzbekistanensis* to infect humans. In the first reported case, the authors postulated that infection might have been acquired through exposure to contaminated bird droppings in Vietnam [6]. Publicly available NCBI database records indicate that *N. uzbekistanensis* has been isolated from wine and koumiss samples in China (Figure 3B). Although the environmental source of *N. uzbekistanensis* strain CY11558 could not be determined, inhalation of environmental propagules remains a plausible route of acquisition, consistent with typical transmission pathways of cryptococcal infections [23].

Titan cell formation, a well‐characterized feature of the *Cryptococcus neoformans* /*C. gattii* complex, is associated with immune evasion, dissemination, and increased resistance to antifungal and environmental stress [13, 24]. Within the genus *Naganishia*, titanization was recently reported in a *N. tulchinskyi* strain [3]. Titan cell formation was not observed in CY11558, suggesting that this morphological transition may not be a common adaptive strategy in this species.

Genomic analysis provides a promising avenue for advancing our understanding of this rare pathogen. A high‐quality draft genome of *N. uzbekistanensis* derived from CY11558 has recently been released [7], offering a platform to investigate potential virulence mechanisms, stress response pathways, thermotolerance, and antifungal resistance determinants. Such insights may support the development of improved diagnostic tools and inform targeted therapeutic strategies.

In summary, this case underscores the importance of molecular identification and susceptibility‐guided antifungal therapy when encountering rare fungal pathogens such as *N. uzbekistanensis*. Continued enhancement of diagnostic databases and genomic resources will facilitate earlier recognition and a deeper understanding of this emerging organism, ultimately aiding clinical decision‐making and improving patient outcomes.

## Funding

This work is supported by the Reform and Development Program of Beijing Institute of Respiratory Medicine (Ggyfz202508), the Peking Union Medical College Undergraduate Curriculum Reform Program (2025bkjg032), and the Peking Union Medical College Hospital Talent Cultivation Program (Category C) (UBJ11583).

## Ethics Statement

The ethics committee of the affiliated Beijing Chao‐Yang Hospital, Capital Medical University approved this study (2021‐ke‐501). Written informed consent for publication was obtained from the patient. All procedures complied with institutional guidelines and the Declaration of Helsinki.

## Data Availability

The data that support the findings of this study are openly available in NCBI GenBank at https://www.ncbi.nlm.nih.gov/genbank/, reference number PQ187063.1; PQ187064.1.
